# Association between serum albumin concentration change trajectory and risk of hypertension: a cohort study in China

**DOI:** 10.3389/fcvm.2024.1325899

**Published:** 2024-05-07

**Authors:** Yinxing Liu, Shan Xu, Hongen Chen, Shuhong Dai, Jiejing Hao, Xi Chen, Ji Zhang, Siguo Li, Jun Liu, Fulan Hu, Yanmei Lou, Changyi Wang

**Affiliations:** ^1^ Department of Non-Communicable Disease Prevention and Control, Shenzhen Nanshan Center for Chronic Disease, Shenzhen, Guangdong, China; ^2^ Department of Epidemiology and Health Statistics, School of Public Health, Zunyi Medical University, Zunyi, Guizhou, China; ^3^ Department of Tuberculosis Prevention and Control, Zunyi Center for Disease Control and Prevention, Zunyi, Guizhou, China; ^4^ Department of Biostatistics and Epidemiology, School of Public Health, Shenzhen University Health Science Center, Shenzhen, Guangdong, China; ^5^ Shenzhen Key Laboratory of Molecular Epidemiology, Shenzhen Center for Disease Control and Prevention, Shenzhen, China; ^6^ Department of Health Management, Beijing Xiaotangshan Hospital, Beijing, China

**Keywords:** serum albumin, trajectory of change, hypertension, cohort study, risk

## Abstract

**Background:**

We sought to assess the risk of hypertension based on the trajectory of changes in serum albumin concentrations.

**Methods:**

A total of 11,946 nonhypertension adults aged 30–60 years who underwent at least 3 medical examinations between 2009 and 2016 were included in this study. Group-based trajectory models were obtained for 4 category groups, and logistic regression models were used to estimate odds ratios (ORs) and 95% confidence intervals (CIs) for each category group of serum albumin concentration and the risk of hypertension.

**Results:**

During a mean follow-up period of 4.30 years, 1,537 hypertension events occurred in 11,946 subjects without hypertension. A high stable trajectory of serum albumin concentrations (OR, 0.70, 95% CI, 0.51–0.96) was associated with a significantly lower risk of developing hypertension. The results of the sensitivity analysis of the high stable trajectory (OR, 0.64, 95% CI, 0.43–0.96) remained statistically significant. Subjects with normal weight and those ≥45 years of age had a significantly lower risk of hypertension at moderate increase (*P* = 0.053 or 0.026) and high stable trajectories (*P* = 0.011 or 0.016). In males and overweight subjects, the risk of hypertension was significantly lower in the high stable trajectory (*P* = 0.038 or 0.044).

**Conclusion:**

In this study, we found that moderate increase in serum albumin concentrations and a high stable trajectory were significantly associated with a reduced risk of hypertension in subjects aged ≥45 years and those with normal weight and that high stable serum albumin concentrations were significantly associated with a reduced risk of hypertension in males and overweight subjects.

## 1 Introduction

Hypertension is an independent risk factor for cardiovascular disease; it can lead to a variety of diseases, including stroke, coronary artery disease, aortic aneurysm, renal failure, heart failure, and death. A large screening study based on 17 million adults in China found the prevalence of hypertension to be 37.2% (1). Therefore, it is particularly important to identify predictors of hypertension and to develop policies to prevent the development of cardiovascular disease.

Serum albumin is a unique multifunctional protein that is synthesized by liver parenchymal cells. As the most abundant protein in plasma, serum albumin accounts for approximately 50% of total plasma protein (2). Serum albumin maintains normal permeability of the microvascular wall, reduces blood viscosity, and inhibits platelet aggregation (3, 4). A longitudinal study by Schalk showed that older adults with decreased serum albumin concentrations, even within the normal range, may be at increased risk for cardiovascular disease (5). In addition, a study of 354 patients with essential hypertension showed an inverse correlation between worsening circadian blood pressure and the serum albumin concentration (6). A 4-year longitudinal study in Japan found that a reduced serum albumin concentration was an important predictor of hypertension (7). A cross-sectional study conducted in Norway reported that serum albumin concentrations were positively associated with systolic and diastolic blood pressure in healthy subjects (8). Based on the above findings, it is reasonable to assume that long-term changes in serum albumin concentrations have a differential effect on the development of hypertension. In this study, adults aged 30–60 years who underwent physical examinations at Xiaotangshan Hospital in Beijing from 2009 to 2016 were enrolled to investigate the correlation between trajectories of changes in serum albumin concentrations and hypertension.

## 2 Methods

### 2.1 Study population

This cohort included adults aged 30–60 years who underwent a comprehensive health examination at Beijing Xiaotangshan Hospital between 2009 and 2016 based on ≥3 health examinations and excluded participants with hypertension, cancer, stroke, coronary artery disease, myocardial infarction, renal disease, and liver disease at baseline, with the final 11,946 participants (6,644 males and 5,302 females) constituting the longitudinal cohort of this study.

The study was approved by the Ethics Committee of Beijing Xiaotangshan Hospital (No. 202006), and the study procedures were conducted in accordance with the 1964 Declaration of Helsinki. The requirement for informed consent was waived because only routine health screening data were used for the analysis.

### 2.2 Data collection

Data on the subjects' demographic characteristics (age, sex), medical history and medications were collected by standardized face-to-face questionnaires, while anthropometric, clinical and biochemical parameters were collected by trained physicians and nurses.

Smoking status (current and/or at least 100 cigarettes in lifetime) and alcohol consumption (Drinking alcohol 12 or more times at different times last year, ≥25 g/day for men and ≥15 g/day for women) were recorded. Trained physicians and nurses measured participants' height, weight, resting heart rate, systolic blood pressure and diastolic blood pressure. The subjects' height and weight were measured while they were dressed casually and without shoes. Body mass index was calculated by dividing body weight (kg) by height squared (m^2^). After at least 5 min of rest, the systolic and diastolic blood pressures of the participants' arms were measured three times with an electronic sphygmomanometer (HEM-770AFuzzy, Omron, Japan) in a seated position, with a 2-min interval between each blood pressure measurement.

Venous blood samples were collected after an overnight fast of at least 8 h. Serum albumin was measured using the bromophenol green contrast method (9). Serum uric acid, white blood cell count creatinine (WBC), total cholesterol, triglyceride, high-density lipoprotein cholesterol (HDL-C), and low-density lipoprotein cholesterol (LDL-C) concentrations were measured using an automated biochemistry analyzer (Model 7600; Hitachi, Tokyo, Japan). Fasting plasma glucose was measured by the glucose dehydrogenase method (Merck, Darmstadt, Germany). Alanine aminotransferase (ALT), aspartate aminotransferase (AST) and blood urea nitrogen concentrations were measured with an automated analyzer.

### 2.3 Definitions

Hypertension was defined by any of the following criteria: (1) self-reported physician diagnosis of hypertension, (2) the use of antihypertension medication within the past 2 weeks, or (3) systolic blood pressure ≥140 mm Hg and/or diastolic blood pressure <90 mm Hg (10).

Prehypertension was defined as subjects with systolic blood pressure ≥120 mmHg and <139 mmHg and/or diastolic blood pressure ≥80 mmHg and <89 mmHg (11).

### 2.4 Statistical analysis

Data management and analysis were performed using StataS/E version 15 (StataCorp, TX) R software version 4.0.5 (www.r-project.org). A *P*-value of 0.05 was considered statistically significant.

**Figure 1:**
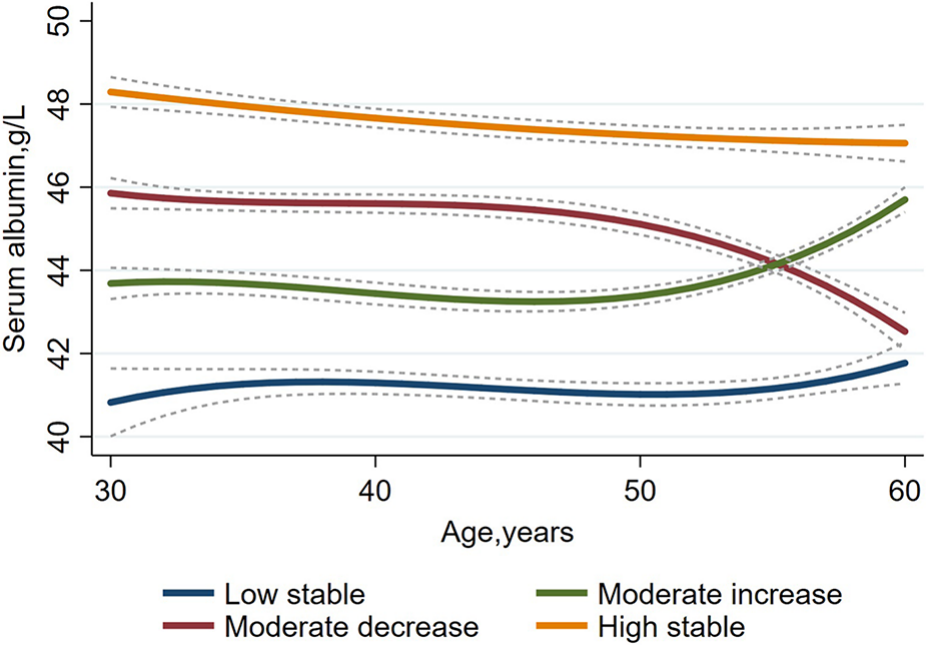
A group-based trajectory model to investigate serum albumin trajectories in a Chinese population aged 30–60 years (*n* = 11,946).

We used group-based trajectory modeling (GBTM) with age as the time scale to explore joint longitudinal changes in serum albumin. The trajectories of study subjects were modeled and grouped using Stata's Proc Traj program (12), a semiparametric mixture model that allows for joint modeling of trajectories for multiple outcomes, which assumes that each participant belongs to only one group and that each group has a different trajectory. Various GBTM models were run before selecting the best model regarding the number of groups and trajectory shapes (constant, linear, quadratic, cubic). First, to determine the optimal number of different groups to describe the heterogeneity in the longitudinal development of serum albumin, various models using 1–7 different groups were fitted, and the model with the least Bayesian information criterion (BIC) (13), mean posterior probability not less than 0.7 and sufficient sample size in each multitrajectory group (>5% of the samples) was selected as the best model, resulting in 4 groups of serum albumin trajectories. Figure 1 shows the four different trajectories of the serum albumin concentration.

Subsequent data analysis was performed using R software, and continuous variables that did not follow a normal distribution were described by the median [interquartile range (IQR)] and analyzed using the Mann‒Whitney *U*-test. Categorical variables were expressed as counts (%) and analyzed using the *χ*^2^ test or Fisher's exact test. Student's *t*-test was used to compare continuous variables across trajectories, and the Kruskal‒Wallis test or *χ*^2^ test was used to compare the incidence of hypertension across trajectory groups. The time to follow-up was defined as the date from first entry into the cohort to confirmation of follow-up or hypertension diagnosis. Odds ratios (ORs) and 95% confidence intervals (CIs) for hypertension were estimated using multivariate logistic regression models with low stability as the reference group to determine differences in the risk of hypertension across trajectory groups.

The covariates included all baseline variables, and three logistic regression models were fitted: Model 1 (unadjusted), Model 2 (adjusted for age, sex, and follow-up time), and Model 3 (further adjusted for smoking, alcohol consumption, diabetes, obesity, body mass index, resting heart rate, systolic and diastolic blood pressure, fasting plasma glucose, triglycerides, total cholesterol, uric acid, blood urea nitrogen, creatinine, LDL-C, HDL-C, WBC, ALT, and AST). In addition, we performed sensitivity analyses excluding subjects who were prehypertension at baseline and excluding subjects diagnosed with hypertension two years before follow-up to assess the robustness of the logistic regression results. We also performed stratified analyses by age, sex, and weight status (normal vs. overweight).

Cumulative average, standard deviation, baseline and end-stage serum albumin concentrations during 2009–2016 were ranked from smallest to largest and divided into five equal quintiles (Q1–Q5). Baseline-stage serum albumin: <42.00, 42.00 to <44.00, 44.00 to <45.10, 45.10 to <47.00, and ≥47.00. Standard deviation serum albumin: <1.516, 1.516 to <2.050, 2.050 to <2.417, 2.417 to <3.000, and ≥3.000. Cumulative average serum albumin: <42.737, 42.737 to <44.033, 44.033 to <44.975, 44.975 to <46.267, and ≥46.267. End-stage serum albumin: <43.00, 43.00 to <45.00, 45.00 to <46.00, 46.00 to <47.00, and ≥47.00. We added details to the study by examining serum albumin concentrations at baseline, cumulative average serum albumin concentrations (cumulative serum albumin concentration from baseline to the last year of follow-up divided by the number of years of follow-up), end-stage serum albumin concentrations in the last year of follow-up, and standard deviations (all available serum albumin concentrations from 2009 to 2016).

## 3 Results

### 3.1 Baseline characteristics of the trajectory groups

The study cohort included 11,946 subjects without hypertension at baseline, aged 42 years (IQR, 36.00–48.00 years), 55.62% of whom were male. A total of 1,537 (12.87%) hypertension events occurred during a mean follow-up period of 4.30 years. Table 1 summarizes the baseline characteristics of subjects in the 4 serum albumin change trajectory groups, including sex, age, smoking, alcohol consumption, obesity rate, body mass index, resting heart rate, systolic blood pressure, fasting plasma glucose, triglycerides, uric acid, blood urea nitrogen, creatinine, LDL-C, HDL-C, WBC, ALT, and AST (ptrend <0.05). Diastolic blood pressure, total cholesterol, and prevalence of diabetes did not differ between the 4 trajectory groups. Supplementary Table S1 demonstrates the baseline characteristics grouped by the occurrence of hypertension events. Age, male proportion, smoking, alcohol consumption, prevalence of diabetes, obesity rate, body mass index, resting heart rate, systolic and diastolic blood pressure, fasting plasma glucose, triglycerides, total cholesterol, uric acid, blood urea nitrogen, creatinine, LDL-C, WBC, ALT, and AST were higher in hypertension patients than in nonhypertension patients (ptrend <0.01), whereas HDL-C concentrations in hypertension patients were significantly lower than those in nonhypertension patients (ptrend <0.001).

**Table 1:** Baseline characteristics of study variables by different trajectories of serum albumin.

Variables	All participants (*n* = 11,946)	Low stable (*n* = 986, 8.25%)	Moderate decrease (*n* = 5,162, 43.21%)	Moderate increase (*n* = 4,665, 39.05%)	High stable (*n* = 1,133, 9.49%)	*P*
Age, years	42 (36–48)	46 (40–52)	41 (35–47)	43 (38–49)	39 (34–46)	<0.001
Male, *n* (%)	6,644 (55.62)	316 (32.05)	3,281 (63.56)	2,197 (47.10)	850 (74.96)	<0.001
Body mass index, kg/m^2^	24.53 (22.39–26.66)	24.00 (22.12–26.39)	24.63 (22.52–26.73)	24.45 (22.30–26.62)	24.71 (22.58–26.84)	<0.001
Resting heart rate, beats/min	73 (70–81)	72 (70–80)	74 (70–81)	72 (70–80)	76 (71–84)	<0.001
Systolic blood pressure, mmHg	111 (105–120)	110 (100–118)	113 (107–120)	110 (102–120)	117 (110–123)	<0.001
Diastolic blood pressure, mmHg	71 (68–78)	70 (67–78)	72 (68–78)	70 (68–78)	72 (68–79)	0.056
Fasting plasma glucose, mmol/L	5.19 (4.90–5.55)	5.11 (4.84–5.46)	5.21 (4.92–5.56)	5.18 (4.89–5.54)	5.24 (4.94–5.58)	<0.001
Triglycerides, mmol/L	1.21 (0.84–1.81)	1.05 (0.76–1.56)	1.27 (0.87–1.90)	1.15 (0.81–1.69)	1.41 (0.96–2.10)	<0.001
Total cholesterol, mmol/L	4.83 (4.25–5.45)	4.84 (4.26–5.55)	4.83 (4.25–5.44)	4.83 (4.26–5.43)	4.81 (4.28–5.47)	0.822
HDL-C, mmol/L	1.34 (1.14–1.57)	1.42 (1.20–1.66)	1.31 (1.12–1.53)	1.36 (1.16–1.60)	1.26 (1.08–1.51)	<0.001
LDL-C, mmol/L	2.94 (2.46–3.43)	2.85 (2.36–3.40)	2.98 (2.49–3.45)	2.90 (2.43–3.40)	3.00 (2.54–3.54)	<0.001
Uric acid, µmoI/L	314.30 (256.40–377.00)	284.00 (236.80–341.50)	325.80 (265.00–386.40)	301.00 (248.80–363.40)	338.10 (279.00–402.60)	<0.001
Blood urea nitrogen, mmol/L	4.7 (3.95–5.59)	4.56 (3.85–5.44)	4.80 (4.00–5.63)	4.64 (3.87–5.50)	4.84 (4.10–5.70)	<0.001
Creatinine, µmoI/L	79.3 (68.7–90.10)	75.30 (68.34–86.05)	81.3 (68.80–91.50)	78.10 (68.88–89.00)	80.00 (68.20–89.72)	<0.001
WBC, 10^9^/L	5.77 (4.89–6.80)	5.60 (4.79–6.59)	5.80 (4.90–6.84)	5.70 (4.83–6.75)	5.90 (5.09–6.90)	<0.001
ALT, U/L	19.00 (14.00–27.60)	16.40 (12.85–22.00)	20.00 (14.00–28.90)	18.00 (13.40–26.00)	22.00 (15.00–32.00)	<0.001
AST, U/L	19.00 (16.30–23.00)	18.40 (16.00–21.57)	19.30 (16.60–23.10)	18.90 (16.10–22.60)	20.00 (17.00–24.00)	<0.001
Current smoker, *n* (%)	2,033 (17.02)	110 (11.16)	993 (19.24)	695 (14.90)	235 (20.74)	<0.001
Alcohol consumption, *n* (%)	3,728 (31.21)	212 (21.50)	1,818 (35.22)	1,248 (26.75)	450 (39.72)	<0.001
Diabete, *n* (%)	617 (5.16)	47 (4.77)	258 (5.00)	252 (5.40)	60 (5.30)	0.756
Obesity, *n* (%)	1,672 (14.00)	119 (12.07)	729 (14.12)	637 (13.65)	187 (16.50)	0.020

Median (interquartile range) unless indicated.

HDL-C, high-density lipoprotein cholesterol; LDL-C, low-density lipoprotein cholesterol; WBC, white blood cell count; ALT, alanine transaminase; AST, aspartate aminotransferase.

### 3.2 Association between the trajectory of change in serum albumin concentrations and the risk of hypertension

**Figure 2:**
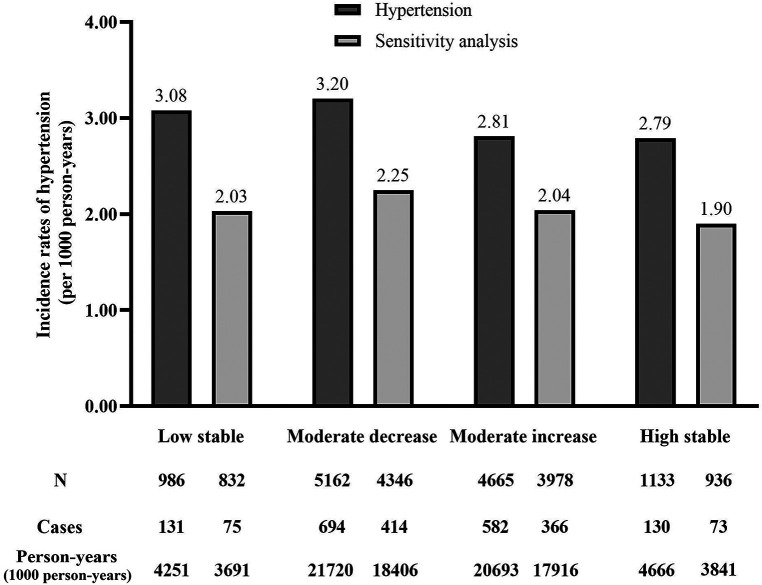
Incidence rates of hypertension in different serum albumin category groups (per 1,000 person-years). Hypertension indicates the annual incidence of hypertension in person-years. Sensitivity analysis indicates the annual incidence of hypertension in person-years after performing sensitivity analysis.

Logistic regression models were used to estimate the association between the 4 serum albumin trajectory groups and the risk of hypertension (Models 1–3; Table 2). In the crude model, the risk of hypertension decreased with a high baseline. High serum albumin levels reduced the risk of hypertension with a low stable trajectory as a control, moderate decrease trajectory (OR, 1.01, 95% CI, 0.83–1.24), moderate increase trajectory (OR, 0.93, 95% CI, 0.76–1.14), and high stable trajectory (OR, 0.84, 95% CI, 0.65–1.09) (*P* = 0.058) (Table 2). After adjusting the model for age and sex (Model 2), the serum albumin high stable trajectory was significantly associated with a reduction in the incidence of hypertension (OR, 0.67, 95% CI, 0.52–0.90). Further adjustment for smoking, alcohol consumption, diabetes, obesity, body mass index, resting heart rate, systolic and diastolic blood pressure, fasting plasma glucose, triglycerides, total cholesterol, uric acid, blood urea nitrogen, creatinine, LDL-C, HDL-C, WBC, ALT, and AST (Model 3) only slightly weakened the association but did not affect the significant association between the serum albumin high stable trajectory and the incidence of hypertension (OR, 0.70, 95% CI, 0.51–0.96) (*P* < 0.001). In the sensitivity analysis, after excluding subjects with prehypertension at baseline, the association between a high stable trajectory of serum albumin concentrations and the risk of hypertension remained statistically significant in the fully adjusted model (OR, 0.66, 95% CI, 0.44–0.98) but was not significant in the moderate decrease and moderate increase trajectories. Figure 2 depicts the person-year incidence of hypertension before and after the sensitivity analysis was performed.

**Table 2:** Association between serum albumin change trajectory and risk of hypertension.

	Low stable	Moderate decrease	Moderate increase	High stable	*P* *
*n* (cases)	986 (131)	5,162 (694)	4,665 (582)	1,133 (130)	
Person-years	4,251	21,720	20,693	4,666	
Incidence (per 1,000 person-years)	3.08	3.20	2.81	2.79	
Model 1^a^	1.00 (ref)	1.01 (0.83–1.24)	0.93 (0.76–1.14)	0.84 (0.65–1.09)	0.058
Model 2^b^	1.00 (ref)	0.85 (0.69–1.06)	0.90 (0.73–1.12)	**0.67** (**0.52–0.90)**	0.061
Model 3^c^	1.00 (ref)	0.93 (0.74–1.19)	0.96 (0.76–1.22)	**0.70** (**0.51–0.96)**	0.091
**Sensitivity analysis** (*n* = 10,092)^d^					
*n* (cases)	832 (75)	4,346 (414)	3,978 (366)	936 (73)	
Person-years	3,691	18,406	17,916	3,841	
Incidence (per 1,000 person-years)	2.03	2.25	2.04	1.90	
Model 1^a^	1.00 (ref)	0.98 (0.81–1.20)	0.97 (0.77–1.22)	0.72 (0.53–1.01)	0.355
Model 2^b^	1.00 (ref)	0.81 (0.63–1.03)	0.91 (0.76–1.24)	**0.59** (**0.35–0.89)**	0.312
Model 3^c^	1.00 (ref)	0.91 (0.68–1.24)	1.05 (0.79–1.42)	**0.64** (**0.43–0.96)**	0.428

Data are odds ratios (ORs) and 95% confidence intervals (CIs).

^a^ Crude model.

^b^ Adjusted for age, sex, and follow-up time.

^c^ Adjusted for covariates in Model 2 as well as smoking, alcohol consumption, diabete, obesity, body mass index, resting heart rate, systolic and diastolic blood pressure, fasting plasma glucose, triglycerides, total cholesterol, uric acid, blood urea nitrogen, creatinine, LDL-C, HDL-C, WBC, ALT, and AST.

^d^ Excluding participants with pre-hypertension at baseline.

* *P *< 0.05 indicates a statistically significant difference in the association between different serum albumin trajectories and risk of hypertension, and *P* ≥ 0.05 indicates no statistically significant difference.

Bolded text in this table indicates that the data are statistically significant.

### 3.3 Association between the cumulative average serum albumin during 2009–2016 and the risk of hypertension

In the crude model, the risk of hypertension decreased with an increase in cumulative mean serum albumin. High serum albumin levels reduced the risk of hypertension, using Q3 as a control, Q1 (OR, 1.30, 95% CI, 1.10–1.54), Q2 (OR, 1.08, 95% CI, 0.91–1.28), Q4 (OR, 1.03, 95% CI, 0.87–1.23) and Q5 (OR, 1.02, 95% CI, 0.86–1.21) (ptrend = 0.278) (Table 3). After adjusting the model for age and sex (Model 2), high cumulative mean serum albumin levels remained significantly associated with a reduction in the incidence of hypertension. Further adjustment for smoking, alcohol consumption, diabetes, obesity, body mass index, resting heart rate, systolic and diastolic blood pressure, fasting plasma glucose, triglycerides, total cholesterol, uric acid, blood urea nitrogen, creatinine, LDL-C, HDL-C, WBC, ALT, and AST (Model 3) only slightly weakened the association but did not affect the significant association between cumulative mean serum albumin. Significant associations between cumulative mean serum albumin and the occurrence of hypertension were Q1 (OR, 1.07, 95% CI, 0.87–1.31), Q2 (OR, 0.96, 95% CI, 0.78–1.17), Q4 (OR, 0.86, 95% CI, 0.71–1.06) and Q5 (OR, 0.74, 95% CI, 0.60–0.92) (ptrend = 0.001). With each 1-SD increase in cumulative mean serum albumin, the risk of hypertension decreased by 8% (OR, 0.92, 95% CI, 0.88–0.96). In the sensitivity analysis, after excluding subjects with prehypertension at baseline, the association between the cumulative mean serum albumin concentration and the risk of hypertension remained significant in the fully adjusted Model Q5 (OR, 0.58, 95% CI, 0.42–0.81) (ptrend = 0.008), with each 1-SD increase in cumulative mean serum albumin associated with a 15% reduction in the risk of hypertension (OR, 0.85, 95% CI, 0.77–0.90).

**Table 3:** Association between cumulative average serum albumin during 2009–2016 and risk of hypertension.

	Cumulative average serum albumin during 2009–2016					*P*	Per 1-SD increase
	Q1	Q2	Q3	Q4	Q5		
*n* (cases)	2,389 (286)	2,389 (360)	2,389 (306)	2,398 (295)	2,381 (290)		11,946 (1,537)
Person-years	11,804	9,670	10,562	10,257	9,042		51,335
Incidence (per 1,000 person-years)	2.42	3.72	2.90	2.88	3.21		3.03
Model 1^a^	**1.30 (1.10–1.54)**	1.08 (0.91–1.28)	1.00 (ref)	1.03 (0.87–1.23)	1.02 (0.86–1.21)	0.278	0.98 (0.94–1.02)
Model 2^b^	1.05 (0.88–1.25)	0.91 (0.76–1.09)	1.00 (ref)	**0.81** (**0.68–0.98)**	**0.69** (**0.57–0.83)**	**<0**.**001**	**0.90** (**0.87–0.94)**
Model 3^c^	1.07 (0.87–1.31)	0.96 (0.78–1.17)	1.00 (ref)	0.86 (0.71–1.06)	**0.74** (**0.60–0.92)**	**0**.**001**	**0.92** (**0.88–0.96)**
**Sensitivity analysis** (*n* = 10,092)^d^	1.17 (0.85–1.60)	1.04 (0.77–1.42)	1.00 (ref)	0.82 (0.61–1.13)	**0.58** (**0.42–0.81)**	**0**.**008**	**0.85** (**0.77–0.90)**

Data are odds ratios (ORs) and 95% confidence intervals (CIs).

^a^ Crude model.

^b^ Adjusted for age, sex, and follow-up time.

^c^ Adjusted for covariates in Model 2 as well as smoking, alcohol consumption, diabete, obesity, body mass index, resting heart rate, systolic and diastolic blood pressure, fasting plasma glucose, triglycerides, total cholesterol, uric acid, blood urea nitrogen, creatinine, LDL-C, HDL-C, WBC, ALT, and AST.

^d^ Adjusted for covariates in Model 3 and further excluding participants with pre-hypertension at baseline.

Bolded text in this table indicates that the data are statistically significant.

### 3.4 Stratified analysis

Stratified analyses were performed by sex, age, and whether or not subjects were overweight. After full adjustment of the model, subjects with normal weight and those ≥45 years of age had a significantly lower risk of hypertension at moderate increase (*P* = 0.053 or 0.026) and high stable trajectories (*P* = 0.011 or 0.016). In males and overweight subjects, the risk of hypertension was significantly lower in the high stable trajectory group (*P* = 0.038 or 0.044), using a low stable trajectory as a reference. Females and age <45 years did not significantly reduce the occurrence of hypertension in any of the trajectory groups (Table 4). The incidence of hypertension was significantly higher in males, subjects aged ≥45 years, and overweight subjects than in females, subjects aged <45 years, and normal weight subjects (Supplementary Table S2).

**Table 4:** Association between serum albumin change trajectory and risk of hypertensive stratified by sex, age and BMI.

	*N* (cases)	Low stable	Moderate decrease	Moderate increase	High stable	*P*
Male	6,644 (1,126)	1.00 (ref)	0.87 (0.63–1.22)	0.89 (0.63–1.25)	**0.68** (**0.46–0.98)**	0.114
Female	5,302 (411)	1.00 (ref)	0.93 (0.64–1.34)	1.05 (0.75–1.49)	0.58 (0.27–1.08)	0.728
Age <45 years	7,196 (757)	1.00 (ref)	1.10 (0.70–1.80)	1.24 (0.79–2.02)	0.86 (0.50–1.48)	0.636
Age ≥45 years	4,750 (780)	1.00 (ref)	0.85 (0.64–1.14)	**0.76** (**0.58–1.00)**	**0.57** (**0.37–0.87)**	**0**.**008**
Normal	5,157 (352)	1.00 (ref)	0.71 (0.47–1.05)	**0.64** (**0.43–0.95)**	**0.51** (**0.29–0.88)**	**0**.**014**
Overweight	6,789 (1,185)	1.00 (ref)	0.96 (0.72–1.30)	1.02 (0.77–1.37)	**0.68** (**0.47–0.99)**	0.170

All analyses were adjusted age, sex, smoking, alcohol consumption, diabete, obesity, body mass index, resting heart rate, systolic and diastolic blood pressure, fasting plasma glucose, triglycerides, total cholesterol, uric acid, blood urea nitrogen, creatinine, LDL-C, HDL-C, WBC, ALT, AST, and follow-up time.

Data are odds ratios (ORs) and 95% confidence intervals (CIs).

Bolded text in this table indicates that the data are statistically significant.

**Figure 3:**
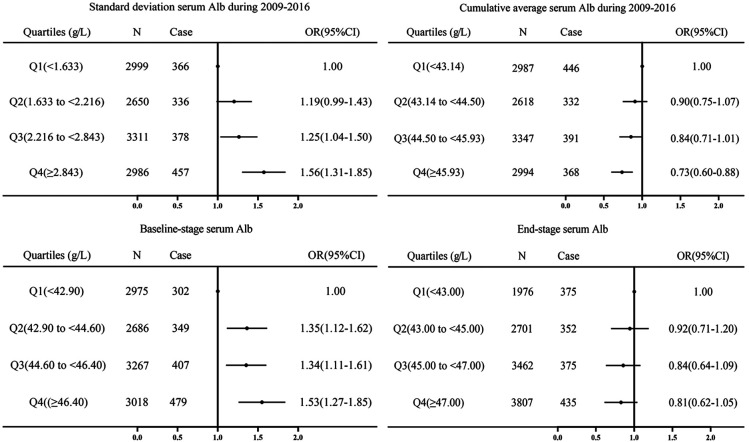
Cumulative average, standard deviation, baseline-stage and end-stage of serum albumin from 2009 to 2016 were divided into quintiles, with Q3 as the control group, and logistic regression adjusted for sex, age, smoking, alcohol consumption, diabete, obesity, body mass index, resting heart rate, systolic and diastolic blood pressure, fasting plasma glucose, triglycerides, total cholesterol, uric acid, blood urea nitrogen, creatinine, LDL-C, HDL-C, WBC, ALT, AST, and follow-up time after posterior odds ratios (ORs) and 95% CIs.

Cumulative average, standard deviation, baseline and end-stage serum albumin concentrations during 2009–2016 were ranked from smallest to largest and divided into five equal quintiles (Q1-Q5), with Q3 as the control. After full adjustment using the model, cumulative average serum albumin values were associated with increased hypertension prevalence during 2009–2016 for Q5 (OR, 0.74, 95% CI, 0.60–0.92). With serum albumin standard deviation Q5 (OR, 1.26, 95% CI, 1.04–1.54) (*P* < 0.001). Q1 of serum albumin at baseline was associated with an decreased incidence of hypertension (OR, 0.76, 95% CI, 0.61–0.95) (*P* < 0.001). There was no significant relationship between the end-stage serum albumin concentration and the prevalence of hypertension (Figure 3). Cumulative average, standard deviations, baseline-stage and end-stage serum albumin concentrations were then stratified by sex, age and body mass index (Supplementary Table S3).

The maximum, minimum, baseline-stage, end-stage, cumulative average and standard deviations of serum albumin concentrations from 2009 to 2016 were described as the median (IQR) according to the different trajectory groups. As shown in Supplementary Table S4, there were statistically significant differences (*P* < 0.001) in the maximum, minimum, baseline-stage, end-stage, cumulative average and standard deviations of serum albumin levels in the different trajectory groups.

## 4 Discussion

To our knowledge, this is the first study to examine the trajectories of serum albumin concentrations and the risk of hypertension in China. In this study, we identified different trajectories of serum albumin concentrations in adults by a group-based trajectory modeling approach. The traditional study approach of dividing participants into subgroups based on various characteristics, while usually ignoring population heterogeneity, may lead to inability to accurately identify internal relationships. There may be unusually large individual differences in serum albumin concentration levels and dynamics over time, and therefore dynamic trajectories of serum albumin concentrations may more accurately predict the risk of hypertension.

Overall, in this retrospective cohort study from 2009 to 2016 at Beijing Xiaotangshan Hospital, four different trajectories of serum albumin concentrations were observed in normal adults. Notably, the risk of developing hypertension was significantly lower in the high stable trajectory groups compared to the low stable group. This association was independent of sex, age, and whether overweight, and remained significant in male, age ≥45 years, normal weight, and overweight subjects. Sensitivity analyses revealed an association between high stable trajectory and prehypertension after full adjustment for covariates, with increases in the standard deviation of serum albumin concentrations and baseline serum albumin concentrations significantly associated with an increased probability of developing hypertension and increases in the cumulative mean of serum albumin concentrations significantly associated with a decreased probability of developing hypertension.

The cumulative incidence of hypertension in the study subjects was 12.87%, similar to the 9.77% observed in Liaoning, China, over a period of 4–6 years (14). The results of this study suggest that the incidence of hypertension is lower when serum albumin is in the high stability group. Previous cross-sectional studies have described the association between serum albumin and the risk of hypertension, and it has been suggested that serum albumin concentrations have been identified as protective factors against cardiovascular disease and are involved in several biologically active processes, such as maintenance of blood colloid permeation, transport and binding of various metabolites *in vivo*, and extracellular oxidative defense (2, 15). It has been suggested that serum albumin may influence blood pressure through a positive correlation mechanism with potassium concentration, which leads to a negative correlation between serum albumin and aldosterone (16). A positive relationship between albumin and potassium may inhibit the renin–angiotensin–aldosterone system, thus preventing hypertension development (17). There are also studies suggesting that the association between serum albumin and blood pressure may be linked to the binding of serum albumin to tryptophan, which has been shown to lower blood pressure (18–22), but tryptophan has not been shown to have a significant effect on blood pressure in other studies (23). It has also been suggested that serum albumin concentrations may affect intravascular osmotic pressure, which in turn affects blood pressure (24). At the same time, previous longitudinal studies have described an association between serum albumin trajectories and risk of hypertension. A five-year retrospective follow-up study in Japan found a positive association between serum albumin and serum potassium and a negative association with the development of hypertension (16). In a longitudinal cohort study in Korea, hypoalbuminemia was an important predictor of early hypertension progression (25).

In this study, our analysis of sex differences showed that the relationship between the four serum albumin concentration trajectories and hypertension varies by sex. Specifically, male subjects were significantly more likely to have hypertension than female subjects, and the incidence of hypertension was significantly lower in the high stable trajectory group, using the low stable trajectory as a reference group. Although hypertension occurs in both males and females, it tends to be significantly higher in males than in females in the same age group (26, 27). A longitudinal study of 3,872 participants in Japan found that for every standard deviation increase in the serum albumin concentration, the risk of hypertension decreased in both males and females (7). Our study showed that the trajectory of serum albumin concentrations was only associated with the incidence of hypertension in males. We found a higher prevalence of hypertension in subjects ≥45 years of age and a stronger association between moderate increase and high stable trajectories and hypertension risk in subjects ≥45 years of age, indicating a significant decrease in hypertension prevalence. In humans, serum albumin levels in both males and females peak at age 20 and begin to decline with age, according to a cross-sectional study in the UK (28), the difference between males and females may be hormonal. The results of this study showed that in normal weight subjects, there was a difference between moderate increase and high stable serum albumin level trajectories and a low incidence of hypertension, while among overweight subjects, there was a difference between a high stable trajectory and a low incidence of hypertension. People who are classified as overweight are more likely to have high blood pressure than those who are not. From 2009 to 2016, the cumulative mean and annual increases in serum albumin concentrations increased, and these increases were associated with the incidence of hypertension. A trend study conducted in Japan between 1980 and 2010 showed a 1.5-fold increase in the effect of being overweight on high blood pressure compared to normal weight people (29). In a study of 131,395 Asian adults, overweight was associated with high blood pressure in all age groups (30), which is consistent with the results of the current study. Overweight and obesity have been linked to several diseases, including chronic inflammation (31). In addition, inflammation may be a major cause of the decrease in serum albumin concentrations (32). Since inflammation is one of the known pathophysiological mechanisms of hypertension, one theory is that low serum albumin is associated with hypertension.

Although the underlying mechanisms linking serum albumin trajectories to the development of hypertension have not been fully elucidated, this finding provides some evidence that the optimal state of serum albumin may be a pattern of high stability within the normal range. Hypertension, as the most important risk factor for cardiovascular disease, causes great physical discomfort to patients and imposes a huge economic burden on patients and their families. Therefore, it is extremely important to propose early interventions for preventive measures against risk factors.

The most important advantage of our study is the large sample size and longitudinal retrospective cohort study design. There are limitations to the study that should be noted. First, the number of patients with hypertension was relatively small during the study period (7 years). Further studies are needed to investigate changes in serum albumin concentrations over time. Second, because the data came from highly educated employees and the proportion of males was high, the study was generally not representative. Therefore, more research is needed in the future to validate our findings.

## 5 Conclusion

In summary, our population-based trajectory modeling approach identified four different trajectories of serum albumin concentrations in Chinese adults. We found that a moderate increase in serum albumin concentrations and a high stable trajectory were significantly associated with a reduced hypertension risk in subjects ≥45 years of age and of normal weight. A high stable trajectory of serum albumin concentrations was significantly associated with a reduced risk of hypertension in both males and overweight subjects.

## Data Availability

The original contributions presented in the study are included in the article/**Supplementary Material**, further inquiries can be directed to the corresponding authors.
