# Supplementary material for: Association between serum albumin concentration change trajectory and risk of hypertension: a cohort study in China
Source: Front Cardiovasc Med. 2024 May 7;11:1325899. doi: 10.3389/fcvm.2024.1325899 (PMC11110567; doi:10.3389/fcvm.2024.1325899)
Supplement: Supplementary file 1 [file Datasheet1.docx]

**Supplementary Table 1** Characteristics at baseline by follow-up outcomes

| Variables | Non-Hypertensives  (N=10409) | Hypertensives  (N=1537) | *P* |
| --- | --- | --- | --- |
| Age,years | 42(36-48) | 45(39-51) | <0.001 |
| Male, n (%) | 5518(53.11) | 1126(73.26) | <0.001 |
| Body mass index,kg/m^2^ | 24.28(22.20-26.39) | 26.22(24.22-28.15) | <0.001 |
| Heart rate,beats/min | 73(70-81) | 76(72-82) | <0.001 |
| Systolic blood pressure,mmHg | 110(104-120) | 120(113-128) | <0.001 |
| Diastolic blood pressure,mmHg | 70(66-77) | 80(75-80) | <0.001 |
| Fasting plasma glucose,mmol/L | 5.17(4.88-5.51) | 5.40(5.07-5.8350) | <0.001 |
| Triglycerides,mmol/L | 1.17(0.81-1.74) | 1.55(1.08-2.30) | <0.001 |
| Total cholesterol,mmol/L | 4.79(4.23-5.41) | 5.05(4.47-5.71) | <0.001 |
| HDL-C,mmol/L | 1.35(1.15-1.58) | 1.25(1.09-1.46) | <0.001 |
| LDL-C,mmol/L | 2.91(2.44-3.40) | 3.13(2.66-3.67) | <0.001 |
| Uric acid,μmoI/L | 308.20(252.00-370.00) | 352.40(292.00-411.60) | <0.001 |
| Blood urea nitrogen,mmol/L | 4.70(3.91-5.52) | 4.95(4.15-5.83) | <0.001 |
| Creatinine,μmoI/L | 78.30(68.10-89.40) | 84.60(75.30-93.50) | <0.001 |
| WBC,10^9^/L | 5.72(4.84-6.75) | 6.05(5.10-7.10) | <0.001 |
| ALT,U/L | 18.30(13.40-26.30) | 23.40(17.00-34.27) | <0.001 |
| AST,U/L | 19.00(16.10-22.60) | 20.50(17.50-25.00) | <0.001 |
| Current smoker, n (%) | 1679(16.13) | 354(23.03) | <0.001 |
| Alcohol consumption, n (%) | 3101(29.79) | 627(40.79) | <0.001 |
| Diabete, n (%) | 458(44.00) | 159(10.34) | <0.001 |
| Obesity, n (%) | 1266(12.16) | 406(26.42) | <0.001 |

median (interquartile range) unless indicated.

Abbreviations: HDL-C, high-density lipoprotein cholesterol; LDL-C, low-density lipoprotein cholesterol; WBC, white blood cell count; ALT, alanine transaminase; AST, aspartate aminotransferase

**Supplementary Table 2**. Hypertensive incidence by serum albumin change trajectory stratified by sex, age and BMI

|  | Low stable | Moderate decrease | Moderate increase | High stable |
| --- | --- | --- | --- | --- |
| Male | 316(21.84) | 3281(16.85) | 2197(17.80) | 850(13.29) |
| Female | 670(9.25) | 1881(7.50) | 2468(7.74) | 283(6.01) |
| P | <0.001 | <0.001 | <0.001 | 0.001 |
| Age < 45 years | 406(6.90) | 3433(11.21) | 2567(10.25) | 790(10.25) |
| Age ≥ 45 years | 580(17.76) | 1729(17.87) | 2098(15.20) | 343(14.29) |
| P | <0.001 | <0.001 | <0.001 | 0.064 |
| Normal | 490(9.59) | 2124(7.02) | 2060(6.07) | 483(6.42) |
| Overweight | 496(16.94) | 3038(17.94) | 2605(17.54) | 650(15.23) |
| P | <0.001 | <0.001 | <0.001 | <0.001 |

Data are n and incidence(%)

**Supplementary Table 3**. Distribution by cumulative average, standard deviation, end-stage and annual increasing rate of serum albumin during 2009-2016 stratified by sex, age and BMI

|  | Male | Female | *P* |
| --- | --- | --- | --- |
| Base-stage serum albumin | 45.00(43.30-47.00) | 44.00(42.00-45.70) | <0.001 |
| Cumulative average serum albumin | 44.86(43.67-46.33) | 44.00(42.57-45.27) | <0.001 |
| Standard deviation of serum albumin | 2.22(1.61-2.75) | 2.22(1.64-2.94) | <0.001 |
| End-stage serum albumin | 46.00(44.00-47.00) | 45.00(43.00-46.00) | <0.001 |
|  | Age < 45 years | Age ≥ 45 years | *P* |
| Base-stage serum albumin | 45.00(43.00-47.00) | 44.00(42.00-45.78) | <0.001 |
| Cumulative average serum albumin | 44.77(43.61-46.33) | 44.00(42.58-45.14) | <0.001 |
| Standard deviation of serum albumin | 2.22(1.53-2.78) | 2.22(1.71-2.89) | <0.001 |
| End-stage serum albumin | 46.00(44.00-48.00) | 45.00(43.00-47.00) | <0.001 |
|  | Normal | Overweight | *P* |
| Base-stage serum albumin | 44.50(42.50-46.20) | 44.60(43.00-46.50) | <0.001 |
| Cumulative average serum albumin | 44.50(43.03-45.83) | 44.50(43.21-46.00) | <0.001 |
| Standard deviation of serum albumin | 2.22(1.66-2.88) | 2.22(1.61-2.81) | 0.015 |
| End-stage serum albumin | 45.00(43.00-47.00) | 45.00(44.00-47.00) | <0.001 |

Data are median (interquartile range).

**Supplementary Table 4.** Distribution of participants in different serum albumin change trajectories by maximum, minimum and annual increasing rate of serum albumin level

|  | Low stable | Moderate decrease | Moderate increase | High stable | *P* |
| --- | --- | --- | --- | --- | --- |
| serum albumin _max_ (g/L) | 43.40(42.30-45.00) | 48.00(47.00-49.00) | 46.00(45.00-47.00) | 50.00(49.00-51.10) | <0.001 |
| serum albumin _min_ (g/L) | 38.00(37.00-39.00) | 43.00(42.00-44.00) | 41.00(39.00-42.00) | 46.00(44.00-47.00) | <0.001 |
| serum albumin _base_ (g/L) | 41.60(40.00-43.00) | 45.40(44.00-47.00) | 43.60(42.00-45.00) | 48.00(46.50-50.00) | <0.001 |
| serum albumin _end_ (g/L) | 42.00(40.00-43.00) | 46.00(45.00-47.00) | 44.00(43.00-46.00) | 49.00(47.00-50.00) | <0.001 |
| serum albumin _average_ | 40.83(40.14-41.37) | 45.45(44.67-46.26) | 43.52(42.67-44.17) | 48.17(47.56-48.84) | <0.001 |
| serum albumin _sd_ | 2.36(1.86-3.00) | 2.17(1.53-2.71) | 2.25(1.73-2.93) | 2.08(1.46-2.65) | <0.001 |

Data are median (interquartile range).
